# Supplementary material for: MRI overestimates articular cartilage thickness and volume compared to synchrotron radiation phase-contrast imaging
Source: PLoS One. 2023 Oct 3;18(10):e0291757. doi: 10.1371/journal.pone.0291757 (PMC10547194; doi:10.1371/journal.pone.0291757)
Supplement: Attachment — Submitted filename: Bairagi.etal.ResponsetoReviews.docx [file pone.0291757.s003.docx]

**We thank both reviewers for their careful consideration of our manuscript, and we feel that the revisions highlighted below improve greatly our submitted revised manuscript.**

Reviewer #1: Accurate evaluation of the morphological characteristics of articular cartilage is essential for the early detection and diagnosis of osteoarthritis (OA). Currently, 3T magnetic resonance imaging (MRI) is widely used to detect OA in clinic. Meanwhile, synchrotron radiation phase-contrast imaging computed tomography (SR-PCI), as a unique imaging technique, is capable of high-resolution imaging of biological soft tissues, including articular cartilage. In this study, the authors evaluated human articular cartilage quantitatively based on MRI and SR-PCI simultaneously. Compared with the non-OA group, the average cartilage thickness and cartilage volume in the OA group were significantly reduced by both imaging methods. In addition, the high resolution SR-PCI is able to map the cartilage surface more accurately than MRI, thus providing more accurate information on cartilage degeneration in OA.

In general, the paper is well written and flows well. I have but a few comments:

1. Keywords- The format of all keywords should be further unified.

**I interpret this as a request that the phrases included as keywords be reduced to single words; in response, we reduced the wording as requested, although a couple of two-word phrases were maintained as keywords (lines 39-40).**

2. Introduction- In addition to MRI and SR-PCI, are there any other common techniques used to conduct imaging studies of articular cartilage? Perhaps the authors can supplement the research status of this part in the introduction.

**In response to this request, we introduced three other synchrotron imaging techniques, providing references for each: “Some synchrotron imaging techniques take advantage of the high resolution, including absorption-based micro-CT, or even revealing material properties by small angle X-ray scattering (36, 37). However, the coherent X-rays in SR can reveal phase contrast depending on the refractive index of different tissues, and diffraction-enhanced imaging is even possible (38).” (lines 79-83).**

3. Figure 2- Can the authors add corresponding scalebars to each panel in Figure 2?

**We apologize for this oversight. Scalebars were added to all images in all figures, where possible. Unfortunately, images in Fig 1 were taken with a phone, so adding scale bars was not possible.**

4. Figure 3- The background of Figure 3H should be consistent with other subgraph images.

**We hope that the revised figure (or figure file type) fixes any inconsistent background noticed in 3H.**

5. Figure 5- Even if there is no statistical significance, it is suggested that the author should also give the corresponding P value to facilitate readers' better understanding.

**In the revised manuscript, non-significant p values are listed.**

6. The author mentions in the discussion that histological preparations might be a good gold standard, but they also have a measurement error due to processing, which may cause shrinkage due to specimen dehydration or other structural changes that occur during embedding, sectioning, and staining of the tissues. Since histology is the gold standard, should the authors consider including histological results for comparison with SR-PCI and MRI results?

**In response to this comment, we included a representative histology image in the revised manuscript (SupFig 2), making careful attempts to correlate the section with a specific virtual slice of the 3D reconstructed PCI dataset. As summarized in Results: “Histological Safranin O staining of one sample confirmed the identity of cartilage in the segmented region and also provided validation of the thickness measures” (lines 202-203); and in Discussion: “While imperfect as a gold standard, our histological sections provided further support for the articular cartilage measures reported here for these imaging modalities” (lines 331-333).**

Reviewer #2: The aim of this manuscript is to compare two imaging methods for cartilage thickness measurement, MRI and synchrotron radiation phase-contrast imaging. The topic is up-to-date as since many years the OA community has been searching for reliable (imaging) biomarkers that would be helpful in disease diagnosis and treatment monitoring. Cartilage thickness has been identified as a potential candidate for a reliable OA biomarker; therefore, it is important to investigate on its properties, specificity and sensitivity. Present study tries to address some of the pitfalls of cartilage thickness measurement from MRI images.

Unfortunately, the results of this study does not provide any considerable answers to the research questions, mostly because of an unfortunate study design. Manuscript contains substantial methodological flaws, which need to be addressed before publication.

1. The MRI part is referred to as “clinical” throughout the manuscript. As the ex-vivo cartilage samples have been examined, the expression “clinical” should be omitted. Using a clinical scanner with the clinical protocol is not sufficient to label this study as clinical.

**While we respectfully disagree that using a clinical scanner with a clinical imaging protocol is not sufficient to label the imaging technique as “clinical”, we agree that the use of dissected tissues might be misleadingly characterized as “clinical”. Therefore, we have removed that term from the title, abstract, and multiple relevant locations throughout the manuscript, as requested.**

2. The choice of SR-PCI as a counter-method for MRI is unclear. Authors even admitted that it could not serve as a gold standard measurement of cartilage thickness. Moreover, it is not mentioned in the manuscript how the slices were matched. What is the slice thickness of SR-PCI images? SR-PCI, despite superior resolution compared to MRI, is also just an approximation of the cartilage thickness measurement. Even on histological slices, it is hard to define the cartilage thickness mostly due to ragged bone-cartilage interface. Any other imaging modality with the lower resolution suffers from inaccuracies in thickness measurement simply because the resolution does not allow seeing those details.

**In response to the comment regarding how the slices were “matched”, we added the following text to the Results section: “Equivalent virtual sections from the same sample were estimated based upon known orientation of the datasets and visual cues from morphology of both the articular cartilage and adjacent subchondral bone.” (lines 175-177); and to the figure legend: “Virtual slices of equivalent regions of the same sample, based upon known orientation of the datasets and visual cues from morphology of both the articular cartilage and adjacent subchondral bone, from 3D reconstructed MRI (C) and SR-PCI (D) datasets…” (lines 387-390).**

**While the slice thickness for SR-PCI images was listed as 27 μm in the Discussion (line 318), we thank the reviewer for this comment, since we should have also added such information to the appropriate location in Methods, which we now did in response to this comment (line 142).**

3. That being said, the title “The Clinical MRI overestimates articular cartilage thickness and volume compared to synchrotron radiation phase-contrast imaging” is misleading, as it implies that SR-PCI is a gold standard method.

**As Reviewer 2 rightly points out in comment #2 above, we acknowledge in our discussion that there is no gold standard method. However, we always try to include the major finding in the title of our papers, and indeed MRI is the current clinical standard for imaging articular cartilage. We could change the title to something more general, like “Comparing articular cartilage measures from MRI and SR-PCI”. On the other hand, we hope that the reviewer appreciates the potential clinical significance of our conclusion that lower-resolution imaging, such as MRI, overestimates articular cartilage health. In this case, we prefer to keep the title as is.**

4. Introduction, last paragraph: here, the research question should be clearly stated. Was it the comparison between two modalities. Or was it a feasibility study for SR-PCI? Please omit the sentences discussing the results: “While both imaging modalities revealed significant differences..”, “These results suggest a specific means to improve..”.

**From this important comment, we appreciate that the main research objectives of this paper, along with the significance relative to previously-published works, were not as clear as they should have been. In response, we changed the final paragraph of the Introduction: “Here, we focussed on two main research objectives. First, can SR-PCI generate sufficient data to quantitate clinically-relevant articular cartilage parameters? Second, how do quantitative image analyses from 3T MRI compare to SR-PCI? Previous proof-of-principle papers using single samples compared SR-PCI to 3T MRI (24, 30, 37, 38). Here, for the first time, measurements of multiple samples of human articular cartilage from medial tibial condyles of non-OA donors and OA patients permit statistical analyses to compare these imaging modalities.” (lines 87-92)**

**While it is common to briefly summarize results at the end of an Intro, also referring to a main discussion point, we have removed those sentences as requested.**

5. Conclusion: Currently it is a compilation of the general statements. Please state the main finding(s) of your study here.

**In response to this comment, we have added two main findings: “Comparing 3T MRI to SR-PCI, both MRI and SR-PCI revealed that the OA group had significantly lower average cartilage thickness and overall cartilage volume, compared to the non-OA group. However, MRI data showed a significantly higher average thickness and overall volume, compared to SR-PCI data, which is likely due to the difference in imaging resolution strengths of the two modalities.” (lines 362-366), and removed the final sentence on longitudinal *in vivo* animal studies.**

6. Another serious issue is the MRI protocol, which is far away from state-of-the art of the protocol for volumetric cartilage analysis (including the thickness measurement). The “OA Research Society International recommendation” to use T1-3D-GRE applies to focal cartilage defect detection, rather than volumetric analysis. On the contrary, the contrast of this sequence is very bad for volumetry analysis due to low contrast between cartilage and a surrounding tissue (bone, fluid effusion, fat) – it is obvious also on the Figure 2 (C). Isotropic 3D-DESS is currently a sequence of choice for any volumetric measurements. Using T1-3D-GRE penalizes the MRI part of this study to a large extent.

**This is a great point. We actually performed a pilot study comparing the DESS to the FLASH (T1-3D-GRE) protocols on one of our samples. To respond to this comment, we included images from this pilot study (Supplemental Figure 1), adding the following description of these results in the Methods section: “Based on a pilot study of one sample comparing T1-weighted 3D fast low-angle shot (FLASH) to dual echo steady state (DESS) MRI protocols, FLASH sequences appeared to give better contrast between cartilage and surrounding tissues and so were used for this study (Supplemental Figure 1).” (lines 117-120). We also added to the Discussion the following speculation to explain what we observed “While 3D-DESS MRI protocols are often a sequence of choice for quantitative measurements of articular cartilage (e.g., (44)), our pilot imaging demonstrated increased contrast between cartilage and surrounding tissues using the FLASH protocol. This unexpected difference might be due to the dissected samples used, instead of larger samples or whole patient limbs. ” (lines 272-276)**

7. It is also recommended to investigate the influence of different available cartilage thickness measurement algorithms (nearest neighbor, surface normal, local thickness, field lines, etc), as they may provide different results for different imaging modalities.

**Comparing how different algorithms could affect the results of quantitative image analyses is an interesting idea, but we unfortunately feel that it is outside the scope of this paper.**

8. In the results section, it is surprising that the MRI-thickness SD is lower compared to SR-PCI (6.8% vs 10% in non-OA and 9.8% vs 14.8%). Is that a mean +/- SD over 4 samples per group? Or is that a mean value through the slices? Either way, considering the superior image resolution of SR-PCI, one would expect much lower SD for SR-PCI measurements. In principle, the cartilage thickness measured here from MRI images includes 3 to 4 pixels from cartilage surface to the bone, at given resolution the accuracy is expected to be rather low.

**This is a great point that we did not appreciate before the reviewer’s comment! Indeed, these SD’s are from the means across 4 samples per group (per imaging modality). In response to this comment, we added the following to the Discussion: “Unexpectedly, despite decreased resolution of MRI, the standard deviations of MRI measures were lower. For example, standard deviation was 10% of the average cartilage thickness for MRI images of OA samples, while it was 15% for SR-PCI images. Given that MRI overestimated measures, compared to SR-PCI, we speculate that partial volume effects contributed to the decreased standard deviation of MRI, especially in thinner regions of articular cartilage.” (lines 306-311)**

9. Please add a representative cartilage segmentation in high detail for both modalities

**In response to this request, we added virtual sections and corresponding segmentation of each imaging modality and experimental group as a Supplemental Figure 2, along with the following clarification in Methods: “Articular cartilage was segmented by semi-automated interactive segmentation using the CT Analyzer software package (SkyScan, Kontich, Belgium; see Supplemental Figure 2 for examples of segmentation).” (lines 149-151).**

10. To be able to discriminate between the accuracies of thickness measurement of these two methods (without having a ground truth), it is recommended to perform test-retest scans (including removing the specimen from the scanner) and inter-reader variability for the semi-automated segmentation.

**Unfortunately, in the absence of test-retest scans or inter-reader data analyses, we had to respond to this comment by including this limitation in our discussion: “Future test-retest scans and evaluating inter-reader variability for the semi-automated segmentation would increase evaluation of accuracies of MRI and SR-PCI in depicting articular cartilage.” (lines 333-335).**

**Although an imperfect solution to the question of accuracy in the imaging modality measurements, we also included a representative histology image, making careful attempts to correlate the sections with specific portions of the MRI and PCI datasets (please see response to Reviewer 1 comment #6 above).**

11. Maybe a subject for a discussion: looking at the Fig 4 and Fig 5, these results suggest that using volume a primary endpoint for the OA detection is much better than using cartilage thickness.

**This very insightful point was incorporated into the Discussion: “At least for the severe cases of OA examined here, these data suggest that overall cartilage volume might be a better diagnostic of articular cartilage health than average cartilage thickness. Regardless of the imaging modality employed, OA measures for average cartilage thickness were 20% lower than non-OA measures. Greater decreases in overall cartilage volume were measured by both imaging modalities, with OA measures over 30% lower than non-OA measures.” (lines 340-344).**
