# Supplementary material for: MRI overestimates articular cartilage thickness and volume compared to synchrotron radiation phase-contrast imaging
Source: PLoS One. 2023 Oct 3;18(10):e0291757. doi: 10.1371/journal.pone.0291757 (PMC10547194; doi:10.1371/journal.pone.0291757)
Supplement: Attachment — Submitted filename: Bairagi.etal.2ndResponsetoReviews.docx [file pone.0291757.s004.docx]

**We thank both reviewers for their careful consideration of our revised manuscript, and we feel that the additional revisions highlighted below improve greatly our submitted re-revised manuscript.**

Reviewer #1: I think authors have revised the manuscript with my comments. The current manuscript has more related information and the mistakes have been solved. I therefore agreed this manuscript can be accepted for publication.

**Many thanks!**

Reviewer #2: The authors' effort put in improving the manuscript is appreciated. Most of the comments have been addressed.

**Thanks for acknowledging our efforts!**

However, there are still couple of remaining issues concerning favoring FLASH over DESS sequence.

1. The first issue is the DESS contrast in Supplemental Figure 1. That does not resemble the contrast of properly optimized DESS. See below the example of the clinical DESS implementation.

Here is the example of a clinical DESS contrast: https://imgtr.ee/image/DESS-clinical.Wb7Fo you see it is substantially different to what is presented in SFig. 1. This should be addressed.

**We thank you so much for highlighting this important issue. In fact, we went back to the original files to make new DESS vs FLASH images, and we found that the previous figure (prepared by a former student) was actually incorrect! I could not open the link for a DESS image provided by the reviewer, but we are confident that the updated DESS image in the revised SupFig1 shows appropriate contrast for properly optimized DESS (e.g., fluid should appear white, and it does).**

The second issue is the different resolution of DESS and FLASH - why is that? Both sequences should be set up in the same way.

**Yes, the sequences were set up the same way, so we hope that the revised comparison reflects similar resolution, as they should be.**

Moreover, after adding the SFig.1, it seems that the FLASH image in the SFig.1 is visually different to the FLASH image in Fig. 2. Please clarify which contrast has been used. If that in the Fig. 2, it certainly disqualifies MRI.

**We hope that the reviewer agrees that the images in the modified figures address this concern. Indeed, we moved revised segmentation images from SupFig2 into revised Fig2, along with the revised image slices, providing a much clearer representation of the data and methodological approaches.**

These issues can falsely lead the readers to the thinking that DESS performs poorly in terms of segmentation as it has worse contrast and resolution than FLASH - and that is not correct.

**From our pilot study, we made a judgement call on whether to follow up on FLASH or DESS, and the wording in the manuscript reflects our interpretation of the pilot images. We might have been incorrect, and we acknowledge this in the Discussion, but we preferred that the FLASH image had more consistent contrast between the cartilage and overlying fluid and subchondral bone (i.e., cartilage had lighter pixels than both the fluid and bone). This is noted in revised lines 272-76, including the sentence “A future study using DESS images would address this potentially influential decision.”.**

Additionally, what is the speculation "This unexpected difference might be due to the dissected samples used, instead of larger samples or whole patient limbs." based on? Why would be the cartilage contrast be different between in-vivo and ex-vivo conditions?

**This sentence was removed.**

2. Referring to the comment #7, I still believe that the different algorithm should be mentioned at least in the discussion as a limitation, as they might contribute to the thickness uncertainty quite a lot.

**For sure, we wrote the following in the Discussion, lines 344-346: “A future study would be useful evaluating the influence of different available cartilage thickness measurement algorithms on similar analyses as performed here.”**
